# Few-Shot Aircraft Door Detection with Visibility-Aware Geometric Regularization

**DOI:** 10.3390/jimaging12100517

**Published:** 2026-10-16

**Authors:** Lexian Liang, Hailong Pei

**Affiliations:** 1 Key Laboratory of Autonomous Systems and Network Control, Ministry of Education, Guangdong Engineering Technology Research Center of Unmanned Aerial Vehicle Systems, South China University of Technology, Guangzhou 510641, China; 18676003383@163.com; 2 Guangzhou Genye Information Technology Co., Ltd., Guangzhou 511436, China

**Keywords:** data-efficient learning, structural constraints, keypoint localization, heatmap regression, attention mechanism, multi-scale feature fusion, occlusion handling, passenger boarding bridge

## Abstract

Aircraft-door detection is a key perception component for automated passenger boarding bridges, but its performance can degrade substantially under limited annotations, illumination changes, partial occlusion, and viewpoint variation. To address this problem, we propose a multi-constraint geometric regularization (MGR) framework for few-shot aircraft-door detection. MGR represents each door using four corner landmarks and a derived center landmark, and explicitly couples appearance learning with door-frame geometry. Specifically, projectively valid diagonal-incidence constraints are combined with visibility-aware and geometry-gated symmetry and orthogonality regularization, while multi-scale RoI feature aggregation and landmark-guided refinement provide complementary appearance cues. Experiments use ResNet-18, ResNet-34, and ResNet-50 with matched target-domain training-instance budgets and support sets, while distinguishing box-only from landmark supervision. With ResNet-50, MGR achieves AP_50_ values of 77.90%, 82.10%, 85.00%, and 86.70% under 15-, 20-, 25-, and 30-shot settings, respectively, giving an average observed margin of 1.10 percentage points over SGFNet, the strongest competing method in the main comparison tables. Rankings are mixed with ResNet-18 and ResNet-34. Ablations indicate additional gains from the combined geometric objective, although refinement contributes a larger observed AP_50_ increase in the ResNet-50 comparison and individual geometric terms are not uniformly beneficial across backbones. These results indicate that explicitly modeling reliable door-frame geometry can provide useful structural supervision for data-efficient aircraft-door detection, particularly under challenging visual conditions.

## 1. Introduction

Locating an aircraft door is a visual perception requirement for automated passenger boarding bridges. Work on task delegation situates bridge automation within a wider airport operating process [1]. Door appearance changes with aircraft livery, illumination, camera position, and obstruction by equipment or the bridge itself. Prior work has investigated cabin-door detection with You Only Look Once version 5 (YOLOv5) and multiple sensors [2]. A detector can provide image-space observations for a docking system, but a bounding box alone does not establish metric clearance or safe control.

This study evaluates single-category aircraft-door detection on a private dataset with diverse aircraft appearances. Sequence-level splitting reduces temporal leakage; dataset composition, metadata coverage, and remaining identity-separation limitations are detailed in Section 4.1.

Few-shot object detection (FSOD) provides adaptation strategies such as the two-stage fine-tuning approach (TFA) [3], Few-Shot Object Detection via Contrastive Proposal Encoding (FSCE) [4], and Decoupled Faster R-CNN (DeFRCN) [5], where R-CNN denotes a region-based convolutional neural network. These methods primarily optimize appearance-driven detection objectives and do not explicitly encode the spatial organization of door landmarks. Under limited supervision, such structural information can provide an additional inductive bias that discourages geometrically implausible landmark configurations. We therefore investigate whether explicit geometric regularization can improve data-efficient aircraft-door detection without assuming that the geometric prior is equally informative under all viewpoints.

The main challenge is to encode geometry without imposing invalid image-space assumptions. Although an aircraft door frame is approximately rectangular in 3D, perspective projection generally maps it to a quadrilateral; image-space right angles and bilateral symmetry are therefore not universally preserved. In contrast, line incidence is projectively invariant. This distinction motivates a formulation that treats diagonal incidence as a robust structural relation, while applying symmetry and orthogonality only as soft, viewpoint-dependent constraints. The formulation also explicitly masks landmarks that cannot be annotated reliably because of occlusion or truncation.

We develop a multi-constraint geometric regularization framework (MGR). The method represents each door using four ordered corner landmarks and a derived center landmark. Diagonal incidence provides the projectively stable constraint, whereas symmetry and orthogonality are modulated by an annotation-derived compatibility gate and visibility masks. A coarse-to-refined landmark pathway couples these constraints to region-of-interest (RoI) features. Annotation-matched ablations assess geometric regularization separately from feature refinement, while comparisons with box-only detectors evaluate the complete system under different supervision.

## 2. Related Work

### 2.1. Detection and Few-Shot Adaptation

The R-CNN family progressed from region-wise feature extraction [6] to shared-image computation in Fast R-CNN [7]. Single-stage alternatives include You Only Look Once (YOLO), its YOLO9000 extension, and the Single Shot MultiBox Detector (SSD) [8,9,10]. Faster R-CNN uses a region proposal network (RPN) followed by region classification and localization [11]. A feature pyramid network (FPN) provides multi-scale representations [12]. These components form the detection backbone of the present design. Meta R-CNN conditions instance-level prediction on support examples [13]. TFA demonstrates the importance of a strong fine-tuning baseline and repeated sampling [3]. FSCE introduces a contrastive proposal objective [4], while DeFRCN addresses interactions between stages and tasks [5]. Their published benchmark scores are not directly comparable with scores on a private aircraft-door dataset. The survey by Köhler et al. [14] organizes the broader few-shot detection literature and its evaluation protocols. Joint few-shot detection and viewpoint estimation also connects recognition to object pose [15]; predicting five door landmarks here does not amount to recovering a calibrated 3D pose.

Our single-category setting differs from conventional base/novel-category FSOD benchmarks. We evaluate target-domain adaptations under the common initialization and data-access protocol specified in Section 4.2, rather than reproducing the methods’ original base-class training protocols.

### 2.2. Structural Priors in Related Visual Tasks

Structural priors have been explored in direct pose estimation [16], structure-aware crack segmentation [17], topology-driven unsupervised keypoint detection [18], and monocular 3D detection [19]. Related work has also used keypoints for fine-grained aircraft recognition [20] and region topology for visual relationship detection [21]. These studies demonstrate the value of explicit structural organization in visual representations, but differ from the present setting in task definition, supervision, and geometric assumptions. Here, the structure is defined entirely in the image plane and is used as a training regularizer for few-shot aircraft-door detection rather than as a calibrated 3D pose model.

### 2.3. Landmark Supervision and Attention

Heatmap-based landmark localization provides spatial supervision beyond bounding boxes, as demonstrated by stacked hourglass networks and simple pose-estimation baselines [22,23]. Distribution-based heatmap supervision has further been studied for pose estimation [24], while geometry-aware few-shot keypoint localization incorporates structural information in label-scarce settings [25]. In contrast to treating all geometric relations uniformly, our formulation separates projectively valid diagonal incidence from approximate image-space rectangle constraints and explicitly handles missing landmark annotations.

The structure-guided few-shot object detection network (SGFNet) [26] incorporates structural guidance, and grammar-guided landmark composition has also been studied for few-shot aircraft detection [27]. Our formulation differs in the geometry encoded for door frames: diagonal incidence is treated separately from approximate symmetry and orthogonality, invisible landmarks are masked explicitly, and the metric constraints are modulated by an annotation-derived compatibility gate. These differences are evaluated through component-level ablations and matched-backbone comparisons.

Channel and spatial attention mechanisms, including squeeze-and-excitation and the convolutional block attention module (CBAM), provide established strategies for feature recalibration [28,29]. In our framework, the multi-scale aggregation and landmark-guided attention modules serve as supporting components rather than standalone methodological contributions. Their effects are therefore evaluated against an annotation-matched landmark-supervised detector so that the contribution of geometric regularization can be isolated from that of additional landmark labels or feature refinement.

## 3. Method

### 3.1. Task Definition and Landmark Convention

**Figure 1:**
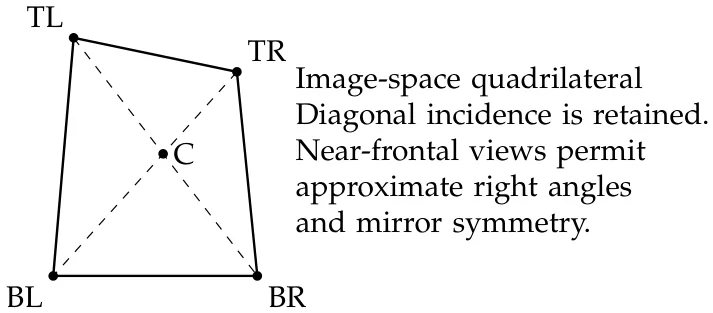
Semantic landmark convention. The four corners define a projected door quadrilateral, and C is the intersection of its two diagonals. The illustration emphasizes that perspective projection need not preserve image-space right angles or bilateral symmetry.

Let *I* denote an input image and *b* a door bounding box. Each door is represented by four ordered corner landmarks ( Figure 1), namely, top left (TL), top right (TR), bottom right (BR), and bottom left (BL), together with a center landmark (C), giving $K^{*}={\left\{k_{i}^{*}\right\}}_{i=1}^{5}$. Left and right follow a consistent upright image annotation convention rather than the physical side of the aircraft. For rounded corners, the landmark is defined by the intersection of the extrapolated straight frame segments. Each corner has an annotation-validity mask $v_{i}\in \left\{0,\;1\right\}$; ambiguous, truncated, or unobservable corners are assigned zero validity. Because C is geometrically derived rather than directly visible, it is considered valid only when all four corner landmarks required to define it are valid. For a specific positive RoI, we further define an effective mask ${\bar{v}}_{i}$ that equals one only when $v_{i}=1$ and the corresponding landmark lies inside that RoI.

The center is defined as the intersection of the two diagonals of the annotated corner quadrilateral rather than as the arithmetic mean of the projected corners. This construction makes diagonal incidence meaningful under a local planar-frame approximation because line incidence is preserved by planar projective transformations [30]. Instances for which the frame is strongly curved, heavily truncated, or visually dominated by a non-coplanar open panel are flagged during annotation and excluded from metric-constraint terms when the landmark geometry is not reliable.

### 3.2. Notation

A superscript ∗ denotes an annotation and a hat denotes a prediction. Variables are defined beside the equations; the Nomenclature before the References, provides the complete symbol list and implementation dimensions.

### 3.3. Region Features and Landmark Refinement

**Figure 2:**
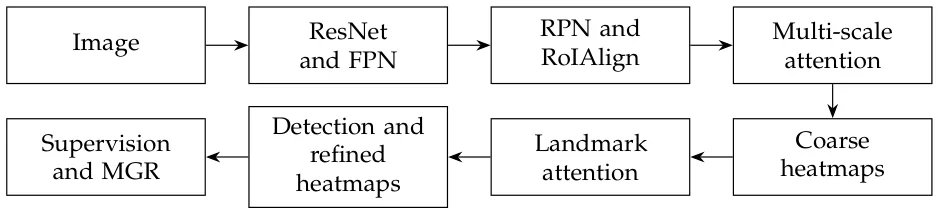
Computation flow of MGR. Geometric constraints regularize the refined landmark predictions during training, while annotation-derived masks and compatibility gates are removed at inference.

As illustrated in Figure 2, the detector follows a Faster R-CNN architecture with a residual network (ResNet) backbone [31], ImageNet initialization [32], and an FPN. Region features are extracted using RoIAlign [33]. For each sampled proposal, RoIAlign produces a 14×14 feature map *X* with 256 channels. Classification and box regression retain the standard two-stage detector formulation, whereas landmark and geometric losses are evaluated only on positive RoIs matched to an annotated door. A landmark target is valid for a positive RoI only when the corresponding annotation is valid and its image-space coordinate lies inside the RoI; otherwise, the RoI-specific landmark mask is set to zero. This prevents out-of-RoI annotations from generating invalid heatmap targets.

Three parallel 3×3 convolution branches with dilation rates r∈{1, 3, 6} produce $Z_{r}$. The following feature-weighting construction uses standard pooling and softmax operations, with channel recalibration and spatial modulation motivated by established attention mechanisms [28,29]. The particular branch weighting and landmark-derived attention used here are specified explicitly rather than reproduced as the equations of those methods. Channel-wise scale weights are obtained from branch features using global average pooling (GAP):

(1) $$a_{r,c}=\frac{\exp h_{c}\left(\mathrm{GAP}\left(Z_{r}\right)\right)}{\sum_{r^{\prime }}\exp h_{c}\left(\mathrm{GAP}\left(Z_{r^{\prime }}\right)\right)},\;X_{m}=X+\sum_{r}a_{r}⊙Z_{r},$$

where *h* is a shared two-layer channel projection with dimensions 256→16→256 and an intermediate rectified linear unit (ReLU), and ⊙ denotes channel-wise multiplication. Here, *r* and $r^{\prime }$ index the dilation branches, *c* indexes feature channels, $h_{c}$ is the channel-*c* output of the shared projection, and $a_{r}$ collects the weights over channels for branch *r*. The operation aggregates multi-scale context within each RoI rather than constructing an additional bottom-up FPN pathway. The branch width and channel-projection dimensions are kept fixed across backbone variants.

A coarse head predicts five 56×56 heatmap logits $H_{i}^{0}$ from $X_{m}$. Their spatial probability distributions are

(2) $$P_{i}^{0}\left(p\right)=\frac{\exp(H_{i}^{0}\left(p\right)/\tau )}{\sum_{q\in \Omega }\exp\left(H_{i}^{0}\left(q\right)/\tau \right)},$$

where *i* indexes landmarks, Ω is the heatmap lattice, *p* is a two-dimensional lattice coordinate, and *q* is a dummy coordinate in the normalization sum. The superscript 0 denotes the coarse head, while 1 denotes the refined head. We set the softmax temperature to τ=1. A bounded landmark-attention map is then formed as

(3) $$A\left(p\right)=\frac{\sum_{i}P_{i}^{0}\left(p\right)}{\max_{q\in \Omega }\sum_{i}P_{i}^{0}\left(q\right)+\epsilon },\;X_{s}=X_{m}⊙\left[1+\gamma \mathrm{Resize}(\mathrm{sg}(A))\right],$$

Here, *A* is the normalized landmark-attention map, γ=1 controls residual modulation, $\epsilon =10^{-6}$ is a numerical stabilizer, and sg denotes the stop-gradient operator. Resize maps the attention map to the spatial resolution of the RoI features, and ⊙ denotes element-wise multiplication with broadcasting over channels. A second landmark head predicts refined heatmaps $H_{i}^{1}$ from $X_{s}$. Each landmark head contains four 3×3 convolutions with 256 channels, followed by bilinear upsampling to 56×56 and a five-channel output layer. The coarse head is supervised directly, whereas its probability maps guide the refinement features without receiving gradients through *A*. The detection head and refined landmark head both operate on $X_{s}$, while the RPN is computed before RoI-level refinement. This coarse-to-refined ordering avoids circular dependence between the final landmark predictions and the features used to produce them.

Coordinates are decoded differentiably using the distribution in Equation (2), separately for each head, following the expectation-based coordinate decoding principle of the differentiable spatial to numerical transform (DSNT) and integral regression [34,35]:

(4) $${\hat{k}}_{i}=\sum_{p\in \Omega }pP_{i}^{1}\left(p\right).$$

The decoded heatmap coordinates are mapped through the same proposal-to-image transformation used to construct the RoI before geometric losses are evaluated. A consistent pixel-center convention is used for target encoding, data augmentation, and inverse mapping, following the coordinate-processing considerations discussed in [36,37]. Because landmark heatmaps are defined in the proposal coordinate system, coordinate decoding uses the associated proposal transformation rather than the subsequently regressed detection box. The predicted landmarks are then retained by proposal index when the corresponding regressed boxes undergo score filtering and non-maximum suppression (NMS). This preserves the heatmap coordinate system, although large box-regression offsets can still introduce misalignment between the final box and its landmarks.

### 3.4. Masked Geometric Regularization

The formulation combines standard point-to-line distance, reflection, and normalized inner-product operations with annotation-validity masking and an annotation-derived compatibility gate. These elementary geometric operations are not claimed as new; the method specifies how they are combined and weighted for door-landmark supervision. The use of incidence is motivated by its preservation under planar projective transformations [30]. Distances are normalized by the ground-truth box diagonal $d^{*}$ during training, giving ${\tilde{k}}_{i}={\hat{k}}_{i}/\left(d^{*}+\epsilon \right)$. Translation cancels in all residuals. Normalizing by a fixed annotation scale prevents the network from reducing a distance penalty merely by inflating its predicted box. Let D(a;b,c) be the squared distance from *a* to the line through b,c:

(5) $$D\left(a;b,c\right)=\frac{{\left[{\left(a-b\right)}_{x}{\left(c-b\right)}_{y}-{\left(a-b\right)}_{y}{\left(c-b\right)}_{x}\right]}^{2}}{{∥c-b∥}_{2}^{2}+\epsilon }.$$

In D(a;b,c), *a*, *b*, and *c* are two-dimensional points, the subscripts *x* and *y* select coordinate components, and ${∥\cdot ∥}_{2}$ denotes the Euclidean norm. The function D(a;b,c) is distinct from the bottleneck dimension *D* used in the complexity analysis. Each residual is weighted by the product of the RoI-specific validity masks of all participating landmarks. The masked mean is normalized by the sum of valid tuple weights plus ϵ and is defined as zero when no valid tuple is available.

**Diagonal incidence.**

With D={(C;TL,BR),(C;TR,BL)}, define

(6) $$L_{\mathrm{inc}}={\mathrm{MaskedMean}}_{(i;j,k)\in D}D\left({\tilde{k}}_{i};{\tilde{k}}_{j},{\tilde{k}}_{k}\right).$$

Each term constrains an independently predicted center relative to a predicted diagonal. Although its annotation is derived from the corners, independent center prediction permits a non-trivial consistency penalty; computing the center from predicted diagonal intersections would satisfy incidence by construction for non-degenerate configurations. Incidence alone is not a complete shape model, as collapsed configurations can also have small residuals, so heatmap supervision remains essential.

**Approximate symmetry.**

Let $u=({\tilde{k}}_{\mathrm{TL}}+{\tilde{k}}_{\mathrm{TR}})/2$, $l=({\tilde{k}}_{\mathrm{BL}}+{\tilde{k}}_{\mathrm{BR}})/2$, and $a=\left(u-l\right)/(∥u-l{∥}_{2}+\epsilon )$. Reflection about the axis through ${\tilde{k}}_{C}$ with direction *a* is

(7) $$R_{a}\left(x\right)={\tilde{k}}_{C}+\left(2aa^{T}-I_{2}\right)\left(x-{\tilde{k}}_{C}\right).$$

Here, *u* and *l* are the upper-edge and lower-edge midpoints, *a* is the stabilized axis-direction vector, *x* is a generic point in normalized coordinates, $I_{2}$ is the 2×2 identity matrix, and ${\left(\cdot \right)}^{T}$ denotes transpose.

The symmetry residual $E_{\mathrm{sym}}$ averages $∥R_{a}\left({\tilde{k}}_{\mathrm{TL}}\right)-{\tilde{k}}_{\mathrm{TR}}{∥}_{2}^{2}$ and $∥R_{a}\left({\tilde{k}}_{\mathrm{BL}}\right)-{\tilde{k}}_{\mathrm{BR}}{∥}_{2}^{2}$. Because the reflection axis depends on all four corners and the derived center, this residual is active only when the complete landmark configuration is valid for the current RoI. The axis accommodates in-plane roll but does not compensate for perspective distortion.

**Approximate orthogonality.**

For each adjacent edge pair (e,f) of the quadrilateral whose vertices are ordered as TL, TR, BR, and BL, including the closing edge from BL to TL, define

(8) $$E_{\mathrm{orth}}=\frac{1}{4}\sum_{\left(e,f\right)}\frac{{\left(e^{T}f\right)}^{2}}{{(∥e∥}_{2}^{2}+{\epsilon )(∥f∥}_{2}^{2}+\epsilon )}.$$

The vectors *e* and *f* are adjacent directed edges formed from the normalized corner coordinates, and the sum includes the four adjacent edge pairs around the ordered polygon. Squaring and normalizing the dot product produces a non-negative, scale-independent penalty that approaches zero as adjacent edges become orthogonal.

**Viewpoint gate.**

Compute $q^{*}=E_{\mathrm{sym}}\left(K^{*}\right)+E_{\mathrm{orth}}\left(K^{*}\right)$ using normalized annotated coordinates. The fixed training gate is

(9) $$g^{*}=\exp\left(-q^{*}/\sigma^{2}\right),\;\sigma =0.25.$$

The gate is set to zero unless the complete corner configuration and the derived center are valid. It measures the compatibility of the annotated image-space quadrilateral with an approximately rectangular projection; it should not be interpreted as an estimate of camera pose. The gate attenuates the metric constraints when the observed shape departs strongly from the frontal-rectangle assumption. We define $L_{\mathrm{sym}}=\mathrm{Mean}\left(g^{*}E_{\mathrm{sym}}\right)$ and $L_{\mathrm{orth}}=\mathrm{Mean}\left(g^{*}E_{\mathrm{orth}}\right)$ over positive RoIs, with invalid configurations contributing zero. Because the normalization uses the RoI count rather than the sum of gate values, small $g^{*}$ values genuinely reduce the contribution of the metric constraints. The gate depends only on annotations and therefore cannot be reduced by the model through its own predictions.

For $q^{*}>0$, smaller σ more strongly attenuates symmetry and orthogonality; incidence is not gated. Section 4.7 evaluates sensitivity around the default σ=0.25.

### 3.5. Joint Objective and Inference

Both landmark heads are trained against normalized Gaussian target distributions $Q_{i}$ of standard deviation 1.5 heatmap pixels, centered at valid RoI-specific landmark targets. For head *h*, use $\ell_{i}^{h}=\sum_{p\in \Omega }Q_{i}\left(p\right)\log\left[\left(Q_{i}\left(p\right)+\epsilon \right)/\left(P_{i}^{h}\left(p\right)+\epsilon \right)\right]$. This is a numerically stabilized Kullback–Leibler divergence from the target distribution to the predicted distribution. The detector losses follow the standard two-stage detection formulation [7,11], while the masking, auxiliary-loss combination, and geometry-weight schedule specify the training objective used in this work. Matching normalized distributions avoids combining bounded Gaussian-logit regression with an almost uniform spatial softmax. Define

(10) $$L_{\mathrm{kp}}=\frac{\sum_{i}{\bar{v}}_{i}\left(\ell_{i}^{1}+0.5\ell_{i}^{0}\right)}{\sum_{i}{\bar{v}}_{i}+\epsilon },\;L_{\mathrm{geo}}=L_{\mathrm{inc}}+L_{\mathrm{sym}}+L_{\mathrm{orth}}.$$

The total objective is

(11) $$L=L_{\mathrm{RPN}}+L_{\det}+L_{\mathrm{kp}}+\lambda \left(t\right)L_{\mathrm{geo}},\;\lambda \left(t\right)=\lambda_{\max}\min\left(1,t/T_{w}\right).$$

Here, $L_{\mathrm{RPN}}$ contains proposal classification and box-regression losses, $L_{\det}$ contains RoI classification and box-regression losses, and $L_{\mathrm{kp}}$ supervises the landmark distributions. The update index is *t*, $T_{w}$ is the geometry warm-up length, and $\lambda_{\max}$ is the final geometry weight. We use $\lambda_{\max}=0.20$ and $T_{w}=200$ steps in the default configuration. The linear warm-up reduces the influence of geometric penalties during early landmark learning. At inference, the network predicts class scores, regressed boxes, and five refined landmark coordinates in a single feed-forward pass. Annotation masks and the compatibility gate are used only during training, so geometric regularization introduces no additional test-time optimization.

The predicted landmarks provide image-space correspondences rather than metric 3D pose. Their use in a passenger-boarding-bridge controller would additionally require calibrated imaging, depth or metric geometry, correspondence validation, and independent system-level safety verification.

### 3.6. Training and Inference Algorithms

Algorithms 1 and 2 summarize the training and inference procedures. Classification uses both foreground and background RoIs, whereas landmark and geometric losses are evaluated only on positive RoIs. For each positive RoI, annotation validity is combined with an in-RoI test to obtain the effective landmark mask; an RoI with no valid landmark target contributes zero auxiliary loss. Parameter updates use adaptive moment estimation with decoupled weight decay (AdamW) [38].

**Algorithm 1** Target-domain adaptation with masked geometric regularization        **Input:** Training images with boxes, $K^{*}$ and *v*; initialized θ; update budget *T*; $\lambda_{\max},T_{w}$.        **Output:** Adapted parameters θ.
    1  **For** t=1,…,T **do**    2         Sample images; jointly transform images, boxes, landmarks and masks.    3         Compute ResNet–FPN features and RPN proposals; match and sample foreground/background RoIs.    4         Extract *X* with RoIAlign for all sampled RoIs.    5         Compute $Z_{r}$, scale-softmax weights $a_{r}$, and $X_{m}=X+\sum_{r}a_{r}⊙Z_{r}$.    6         Predict $H^{0}$ and normalize spatially to obtain $P^{0}$.    7         Form *A* from $P^{0}$; compute $X_{s}=X_{m}⊙\left[1+\gamma \mathrm{Resize}\left(\mathrm{sg}\left(A\right)\right)\right]$.    8         Predict class scores, box offsets, and refined probabilities $P^{1}$ from $X_{s}$.    9         Compute $L_{\mathrm{RPN}}$ and $L_{\det}$ with standard matched targets.  10         On positive RoIs, combine annotation validity with the in-RoI test, construct Gaussian targets for valid landmarks, and compute $L_{\mathrm{kp}}$.  11         Decode $P^{1}$ by expectation; map coordinates to the image and normalize by $d^{*}$.  12         Compute masked diagonal residuals; derive $g^{*}$ from valid annotations and set it to zero when the complete configuration is unavailable.  13         Compute $L_{\mathrm{geo}}=L_{\mathrm{inc}}+L_{\mathrm{sym}}+L_{\mathrm{orth}}$; zero invalid terms.  14         Set $\lambda \left(t\right)=\lambda_{\max}\min\left(1,t/T_{w}\right)$ and assemble Equation (11).  15         Backpropagate and update unfrozen θ using AdamW with learning rate $\eta_{t}$.  16     **End for**; retain the checkpoint with highest validation AP_50_, choosing the later checkpoint in a tie.


**Algorithm 2** Feed-forward detection and landmark inference          **Input:** Image *I*, trained θ, and fixed proposal/score/NMS settings.          **Output:** Retained door boxes, scores, and five image-space landmarks per box.
   1    Resize the image; compute backbone/FPN features and RPN proposals.   2    Apply the fixed proposal cap and RoIAlign to the retained *R* proposals.   3    Compute $X_{m}$, coarse $P^{0}$, attention *A*, and refined features $X_{s}$.   4    Predict box offsets, class scores, and refined heatmaps $P^{1}$.   5    Decode each $P_{i}^{1}$ by expectation and map it through its *proposal* coordinates to the image.   6    Decode boxes; apply score filtering and NMS, retaining the corresponding landmark predictions by proposal index.   7    Undo the input resize and return the retained observations.


Inference preserves the proposal-coordinate decoding and proposal-index association defined above. Annotation masks, compatibility gates, and geometric optimization are not used at inference.

### 3.7. Computational Complexity

We count multiply–accumulate operations (MACs), with one MAC corresponding to approximately two floating-point operations under the multiply-plus-add convention; one giga-MAC (GMAC) denotes $10^{9}$ MACs. Let $C_{\mathrm{base}}\left(I,R\right)$ include the backbone, FPN, RPN, RoI extraction and ordinary detection head. All scale branches preserve *C* channels on *S* spatial positions. Each landmark head contains *L* dense 3×3 convolutions on this grid, followed by bilinear upsampling and a 1×1 projection to *N* heatmaps on *U* positions. The shared channel projection has bottleneck dimension D=C/16. Dilation changes receptive-field coverage without changing the number of kernel weights. The operation and parameter counts below are derived for the explicitly specified architecture by summing the costs of its convolutions and projections; they are analytical calculations for this implementation rather than formulas adopted from another detector.

The leading additional forward cost over the box-only detector is

(12) $$\begin{array}{cc} C_{\mathrm{extra}} & =9RSC^{2}\left(B+2L\right)+2\left(R\cdot U\cdot C\cdot N\right)+2\left(B\cdot R\cdot C\cdot D\right)\\ \; & \;+O\;\left(B\cdot R\cdot S\cdot C+R\cdot U\cdot C+R\cdot N\cdot U\right). \end{array}$$

where the first term comprises scale convolutions and the two landmark heads; the second comprises the output projections; the third is the shared multilayer perceptron (MLP) evaluated for each branch. The products use the dimensions defined in Table A1: *R* is the number of processed RoIs, *U* the heatmap area, *C* the channel count, *N* the landmark count, *B* the number of scale branches, *D* the channel-projection bottleneck dimension, and *S* the RoI grid area. Pooling, weighted fusion, interpolation, spatial normalization and coordinate expectations appear in the lower-order term. Thus, $C_{\mathrm{total}}=C_{\mathrm{base}}+C_{\mathrm{extra}}$; proposal filtering and NMS remain common detector costs. A naive NMS implementation can take $O(R^{2})$ comparisons and is not included in the convolution MAC count.

Ignoring biases, additional learned weights total

(13) $$P_{\mathrm{extra}}=9C^{2}\left(B+2L\right)+2CN+2CD.$$

For C=256, B=3, L=4, D=16, S=196, U=3136, and N=5, the auxiliary pathway contains 6,498,816 learned weights and requires approximately $1.280\times 10^{9}$ leading MACs per processed RoI under this analytical model. With a cap of 100 processed RoIs, the corresponding upper-bound leading cost is approximately 128 GMACs per image. These values are analytical counts rather than profiler measurements and therefore complement, rather than replace, measured latency and memory consumption.

Relative to the annotation-matched two-head keypoint baseline, the scale branches and shared MLP add $9BC^{2}+2CD=1,777,664$ weights; landmark attention adds no learned weights under fixed γ. Relative to the no-geometry model, geometric regularization adds *zero* learned parameters and no inference operations. With a bounded-degree landmark constraint graph, its training residuals cost $O(R_{+}N)$; the present five-point graph has two incidence terms, two reflected pairs and four adjacent-edge terms, hence $O(R_{+})$ cost. Heatmap decoding still costs $O(R_{+}NU)$ and belongs to the landmark pathway. Backpropagation through these local losses changes constants rather than asymptotic order.

A conservative bound on auxiliary training activations, excluding the base detector and optimizer state, is

(14) $$O\;\left(R\left[(B+2L)SC+2UC+2NU\right]\right).$$

During inference, intermediate branch and head buffers can be released; for fixed *B* and *L*, the working activation order is O(R[SC+UC+NU]). Batching RoIs trades peak memory against execution overhead.

## 4. Experiments

### 4.1. Dataset and Data Splits

The private dataset contains 102 aircraft-type labels in its acquisition metadata, while aircraft door is the single foreground detection class. The in-domain training pool contains 3600 images, 3960 door instances, and 360 approach sequences; validation contains 300 images, 330 instances, and 30 sequences. These partitions and the in-domain test set cover three airports. Table 1 summarizes the evaluation subsets. The separate held-out-airport test uses a fourth airport excluded from training, validation, and parameter selection.

Data are partitioned by approach sequence before support sampling. Known aircraft identities are kept within one partition, and near-duplicate frames are removed or assigned to the same partition. Identity metadata are unavailable for 54/360 training sequences (15.00%), 5/30 validation sequences (16.67%), 21/120 in-domain test sequences (17.50%), and 6/36 held-out-airport sequences (16.67%). Thus, all splits are sequence-disjoint, but identity separation cannot be verified for unidentified aircraft.

The main few-shot budgets are K∈{15, 20, 25, 30} annotated door instances, with supplementary 5-shot and 10-shot evaluations. All doors in a selected image are annotated and counted toward *K*. Within each run, support sets are nested across budgets and shared across methods and backbones; validation and test partitions remain fixed. For the 15-, 20-, 25-, and 30-shot budgets, the respective numbers of unique support sequences are (8, 11, 14, 16) in Run 1, (9, 12, 15, 18) in Run 2, and (7, 10, 13, 15) in Run 3. A sequence is counted once within each support set. The 330 validation instances are additional labels outside *K*, so the shot count describes adaptation training rather than total annotation expenditure.

Day and Night are mutually exclusive image-level categories assigned from acquisition records and visual inspection, comprising 65% and 35% of in-domain test images. Occlusion (25%) requires another object to visibly obscure a target door or its frame; shadows, reflections, and image-boundary truncation alone do not qualify. Oblique (20%) requires visually evident perspective foreshortening or convergence of opposing frame edges, excluding in-plane rotation alone; it is not a calibrated angle category. Occlusion and Oblique may overlap with each other and either illumination category. Conditional evaluation includes all annotated doors and predictions in selected images. Sequences are counted once per subset, so overlapping subset counts must not be summed.

MGR requires four corner locations and validity labels in addition to boxes; the center is computed from the corners without independent manual placement. Table 2 reports annotation times in seconds. The additional corner annotation and validity checking take 10 seconds per instance, doubling the box-only annotation time. Equal instance budgets therefore do not imply equal annotation effort.

Four annotators produced the original dataset annotations. Two different annotators, neither involved in the original annotation, independently reannotated 300 door instances spanning illumination, occlusion, viewpoint, and scale variation. Agreement was measured before reconciliation. Coordinate disagreement uses corners valid for both annotators and is reported in original-image pixels and after normalization by the square root of the reference box area; validity agreement uses all 1200 corner pairs. The center is derived separately from each corner configuration. Table 3 reports agreement and invalid-label rates across the four partitions. Its exclusion counts account for overlapping categories: 180+120−30+90=360 instances are excluded from metric terms but retained for box-based detection evaluation.

### 4.2. Implementation Details and Fairness Controls

Meta R-CNN, TFA, FSCE, DeFRCN, SGFNet, MGR (Box-only), and MGR use the same ImageNet-pretrained backbone initialization within each matched-backbone comparison. All remaining modules are initialized without detection-pretrained weights. For these matched-backbone implementations, no external detection dataset or preliminary training on the unused aircraft-door training pool is used. Methods originally designed for base-to-novel transfer are therefore evaluated as single-category target-domain adaptations, not reproductions of their original base-class training protocols.

For the matched-backbone comparisons, adaptation uses AdamW [38] with initial learning rate and weight decay both $10^{-4}$, a 200-update linear learning-rate warm-up, and a single cosine decay segment without restarts [39]. Training lasts 2000 updates with batch size two. The first ResNet stage and batch-normalization statistics are frozen. Images are resized to a short side of 800 pixels and a maximum long side of 1333 pixels. Color jitter and horizontal flipping are applied consistently to images and annotations; flipping exchanges TL/TR and BL/BR. ResNet-18, ResNet-34, and ResNet-50 share the FPN width and RoI-head dimensions.

For MGR and the matched-backbone comparisons, the checkpoint with the highest validation AP_50_ is selected. Ties are resolved in favor of the later checkpoint. This validation-based checkpoint selection is distinct from hyperparameter selection, and test results are not used to choose checkpoints.

The defaults $\lambda_{\max}=0.20$, σ=0.25, γ=1, $T_{w}=200$, and Gaussian target width of 1.5 pixels on the 56×56 heatmap grid were fixed before test evaluation, without a validation-set hyperparameter search, and shared across backbones. The geometry warm-up $T_{w}$ is distinct from learning-rate warm-up. Sensitivity sweeps assess these common defaults rather than select the best test-set configuration.

MGR (Box-only) retains the backbone, FPN, RPN, RoIAlign, and standard detection head, removing both landmark heads, both refinement modules, and geometric losses. Its objective is $L_{\mathrm{RPN}}+L_{\det}$. The Keypoint baseline retains both supervised landmark heads but removes refinement and geometry. No geometry retains refinement and landmark supervision with $\lambda_{\max}=0$. All use the shared initialization, support sets, and adaptation budget. Consequently, Box-only versus Keypoint baseline changes architecture and supervision; Keypoint baseline versus No geometry evaluates refinement; No geometry versus Full isolates the geometric loss.

The supplementary YOLOv5s and full-pool evaluations retain the same validation and test partitions. Full-pool training uses all 3960 training instances. YOLOv5s uses the official training configuration without modification, rather than the AdamW and 2000-update protocol specified above for the matched-backbone experiments. It provides an architecture-specific box-only reference, not a matched-backbone comparison or a reproduction of the complete system in [2]. Full-pool results are empirical references rather than guaranteed upper bounds.

Experiments use an NVIDIA RTX 3090 graphics processing unit (GPU) and Compute Unified Device Architecture (CUDA) 11.3. Runtime comparisons use ResNet-50 at 30 shots and 32-bit floating-point precision (FP32). With the stated image resizing, inference uses batch size one and at most 100 RoIs. Following 50 warm-up passes, latency is averaged over 200 images in the same order for all configurations, with CUDA synchronization at timing boundaries. Timing includes the forward pass, box decoding, and NMS, excluding disk access, CPU preprocessing, and host-to-device transfer. Throughput is the reciprocal of mean serial latency; peak allocated memory is reported in gibibytes (GiB). Training costs use the stated batch size of two. Analytical operation counts are given in Section 3.7.

### 4.3. Metrics and Randomness

Because the task contains one foreground category, detection performance is reported using average precision (AP) at an intersection over union (IoU) threshold of 0.50, denoted AP_50_. Operating-point precision and recall use a score threshold of 0.50, NMS IoU of 0.50, and one-to-one IoU-0.50 matching. Any threshold tuning is performed on the validation set only.

For matched detections, object keypoint similarity (OKS) follows the Common Objects in Context (COCO) formulation [40], adapted to the box-based scale and task-specific tolerance below:

(15) $$\mathrm{OKS}=\frac{\sum_{i}v_{i}\exp[-∥{\hat{k}}_{i}-k_{i}^{*}{∥}_{2}^{2}/\left(2s^{2}\kappa_{i}^{2}\right)]}{\sum_{i}v_{i}+\epsilon },$$

Here, $s=\sqrt{\mathrm{area}(b^{*})}$ and the common tolerance is $\kappa_{i}=0.05$. We report 100× mean OKS, not COCO keypoint AP, and interpret it alongside recall because unmatched doors do not contribute. Unlike COCO’s landmark-specific tolerances, $\kappa_{i}$ is not calibrated from annotator variance: an error of 0.05s contributes exp(−1/2) under this formula. Absolute values therefore describe localization under a fixed task-specific tolerance rather than annotation-calibrated accuracy.

We define geometric consistency rate (GCR) on matched detections for which all four corner annotations and the derived center are valid. A prediction is counted as geometrically consistent when its corner polygon is non-self-intersecting and convex, the predicted center lies inside the polygon, and the center-to-diagonal distance is below $0.02d^{*}$ for both diagonals. GCR measures internal configuration plausibility rather than agreement with the ground-truth door outline and is therefore used only as a complementary diagnostic.

Each configuration uses three paired support-set/training runs. Tables report means and sample standard deviations (SDs). For the main comparisons, the paired AP_50_ difference in run *i* is $d_{i}={\mathrm{AP}}_{50}^{\mathrm{MGR},i}-{\mathrm{AP}}_{50}^{\mathrm{baseline},i}$, with two-sided 95% confidence interval (CI) $\bar{d}\pm t_{0.975,n-1}s_{d}/\sqrt{n}$, where n=3 and $s_{d}$ is the sample SD of the differences. Positive differences favor MGR. These are individual, unadjusted intervals, not simultaneous evidence across all comparisons. Run-to-run variation combines support sampling and optimization; it does not separately estimate their variance contributions. Ablation and sensitivity differences are interpreted descriptively unless a corresponding paired interval is reported.

### 4.4. Main Comparison

Table 4, Table 5 and Table 6 show that MGR achieves competitive mean AP_50_, with performance differences depending on backbone capacity and shot budget. With ResNet-50, MGR exceeds SGFNet in mean AP_50_ by 1.80, 1.40, 0.50, and 0.70 percentage points at 15, 20, 25, and 30 shots, respectively. The average observed difference is 1.10 points, compared with 2.38 points relative to DeFRCN. The margins are smaller with the lighter backbones. Relative to SGFNet, the average mean difference is approximately 0.07 percentage points for both ResNet-18 and ResNet-34. SGFNet has higher mean AP_50_ in four of the eight settings across these two backbones. In particular, MGR obtains 85.71% AP_50_ with ResNet-34 at 30 shots, compared with 85.77% for SGFNet, giving a difference of −0.06 percentage points. These averages summarize the observed mean differences across shot budgets and do not establish statistically reliable superiority across all settings.

Comparisons with box-only methods involve differences in target supervision and model design. The contribution of geometric regularization is therefore assessed using the annotation-matched Full versus No geometry comparison. At 30 shots, adding geometry changes mean AP_50_ from 85.09% to 86.70% with ResNet-50 and from 83.51% to 84.07% with ResNet-18, corresponding to observed differences of 1.61 and 0.56 percentage points.

Table 7 distinguishes the computational cost of the complete architecture from the incremental cost of geometric regularization. Relative to the Box-only baseline, MGR increases inference latency from 52.0 to 122.0 ms per image and peak inference memory from 1.92 to 3.35 GiB, corresponding to increases of 134.6% and 74.5%, respectively. Its serial inference throughput is 8.20 images/s under the stated timing protocol. The additional cost reflects the landmark-prediction and feature-refinement pathway, which remains active during inference.

No geometry and MGR have the same inference architecture because the geometric losses and annotation-derived gate are used only during training. Their inference latency and peak allocated memory are identical at the reported precision. During training, enabling geometry increases the average update time from 346.0 to 359.0 ms, an increase of 3.8%, and peak allocated memory from 7.60 to 7.82 GiB, an increase of 2.9%. Relative to the Box-only baseline, the complete MGR configuration increases training time per update by 95.1% and peak training memory by 62.9%. Thus, the geometric loss introduces a comparatively small incremental training cost within the landmark-supervised architecture, whereas the complete system has appreciable overhead relative to box-only detection.

Table 8 extends the comparison to 5 and 10 training instances. MGR exceeds SGFNet in mean AP_50_ by 0.86 and 1.25 percentage points, respectively. Relative to the annotation-matched No geometry configuration, the corresponding differences are 1.33 and 1.72 points, compared with 1.82 points at 15 shots. The observed geometry benefit therefore does not increase monotonically as the training budget decreases. These comparisons describe mean performance changes and do not establish statistically reliable superiority from three runs alone. The additional 330 validation instances remain outside the shot count, so these results do not represent a total labeling budget of only 5–15 instances.

Table 9 shows 30-shot to full-pool gaps of 8.36, 8.19, and 5.73 AP_50_ points for YOLOv5s, MGR (Box-only), and MGR. The MGR margin over Box-only narrows from 3.31 points at 30 shots to 0.85 points with the full pool. These differences concern the complete landmark-supervised architecture, not geometry alone, and show the remaining accuracy gap alongside the additional labeling requirements.

### 4.5. Condition-Specific Comparisons

Table 10 shows that the relative performance depends on the evaluation condition. Compared with SGFNet, MGR has observed mean AP_50_ margins of 0.81, 0.87, and 0.44 percentage points under nighttime, occlusion, and held-out-airport conditions, respectively, but lower mean AP_50_ by 0.34 and 0.69 points under daytime and oblique conditions. The nighttime and occlusion margins are smaller than the corresponding differences of 1.89 and 3.08 points relative to DeFRCN.

We compute offline gate diagnostics from positive RoIs of the three 30-shot ResNet-50 checkpoints, using the training matching rule before refinement. Table 11 separates the valid-configuration fraction from the conditional distribution of $g^{*}$, excluding mask-induced zeros from the latter. Evaluation annotations are used only for this diagnostic, not inference. The oblique median is 0.07 vs. 0.64 for Day, and the respective fractions with $g^{*}<0.1$ are 61.8% and 12.6%, indicating attenuation of metric constraints. Occlusion has a lower valid fraction (63.7%), illustrating the separate role of masking. These diagnostics do not establish a detection benefit under oblique views, where MGR remains below SGFNet, FSCE, and DeFRCN.

### 4.6. Single-Factor Ablations

As shown in Table 12 and Table 13, each “No” row changes one component relative to Full. “No geometry” sets $\lambda_{\max}=0$; “No multi-scale attention” replaces the multi-scale branch with the identity mapping; and “No landmark attention” bypasses the multiplicative landmark-attention map while retaining both supervised landmark heads. “No incidence”, “No symmetry”, and “No orthogonality” remove only the corresponding penalty term without renormalizing the remaining terms. Because $g^{*}$ is computed from annotation symmetry and orthogonality, these two rows isolate the penalty contribution but do not remove the corresponding annotation information from the gate. “No viewpoint gate” sets $g^{*}=1$ for otherwise valid configurations, and “No warm-up” applies the full geometry weight from the first update. The keypoint baseline removes both feature-refinement modules and all geometric terms while retaining the two supervised landmark heads; it is therefore an annotation-matched reference rather than a single-factor ablation. Removing all geometric regularization changes AP_50_ by 1.61 points for ResNet-50 and 0.56 points for ResNet-18.

The symmetry term does not improve every reported mean. With ResNet-18, No symmetry obtains 84.11% AP_50_ compared with 84.07% for Full, whereas Full exceeds No symmetry by 0.31 percentage points with ResNet-50. These small differences do not establish either a reliable benefit or a reliable degradation. Nevertheless, approximate image-space symmetry can introduce an inappropriate bias under perspective distortion, and compatibility gating need not eliminate this effect completely. We therefore regard symmetry as a potentially useful but condition-dependent regularizer rather than a uniformly beneficial constraint.

The results also illustrate an important distinction between geometric plausibility and localization accuracy: disabling the gate can increase GCR while reducing AP and OKS. A stronger rectangle bias may produce more regular configurations even when those configurations fit perspective-distorted doors less accurately. Consequently, GCR is interpreted jointly with detection and landmark-localization metrics rather than combined into an ad hoc composite score.

Table 14 separates operating-point precision and recall from the integrated AP metric. The highest precision at a fixed threshold need not correspond to the highest AP. All operating-point comparisons therefore use the same matching rule and a score threshold determined without access to the test labels.

Geometry–refinement interaction.

We conduct a two-factor ablation with ResNet-50 at 30 shots, treating multi-scale aggregation and landmark-guided attention jointly as the refinement factor and the combined geometric loss as the geometry factor. All four configurations retain both supervised landmark heads and identical landmark annotations, support sets, paired seeds, and training settings. When refinement is disabled, both landmark heads and the detection head use the original RoI features without multi-scale aggregation or landmark-guided modulation. The Geometry only configuration retains the full geometric objective and its default gating and warm-up settings while disabling both refinement modules.

Table 15 shows observed AP_50_ increases of 1.48 and 1.61 percentage points from geometric regularization without and with refinement, respectively. Conversely, refinement increases AP_50_ by 1.83 points without geometry and 1.96 points with geometry. Refinement therefore contributes larger mean improvements in these comparisons, while geometry provides an additional improvement in both settings. The difference between the two conditional geometry effects is 0.13 points, which does not by itself establish a statistically reliable interaction. We consequently distinguish the methodological focus on geometric regularization from a claim that it is the dominant source of detection improvement.

Independent center prediction.

Using ResNet-50 at 30 shots, we compare Full and No incidence with a four-corner variant under the same support sets and training settings. The variant predicts four heatmaps per head, constructs attention from those maps, and computes the center from predicted diagonals for symmetry during training and as an inference output. It removes center supervision and incidence loss while retaining the other components. Full versus No incidence isolates the incidence penalty; the four-corner comparison changes both the representation and attention inputs.

Table 16 reports mean AP_50_ values of 86.08%, 86.21%, and 86.70% for Four-corner, No incidence, and Full, respectively. The 0.13-point difference between Four-corner and No incidence does not establish a reliable advantage from independent center prediction alone, particularly because the attention inputs also change. Within the five-landmark architecture, adding incidence regularization yields an observed increase of 0.49 points. The comparison supports evaluating the center as an auxiliary consistency target, but does not establish that an independently predicted fifth landmark is universally necessary.

### 4.7. Sensitivity and Qualitative Evaluation

**Figure 3:**
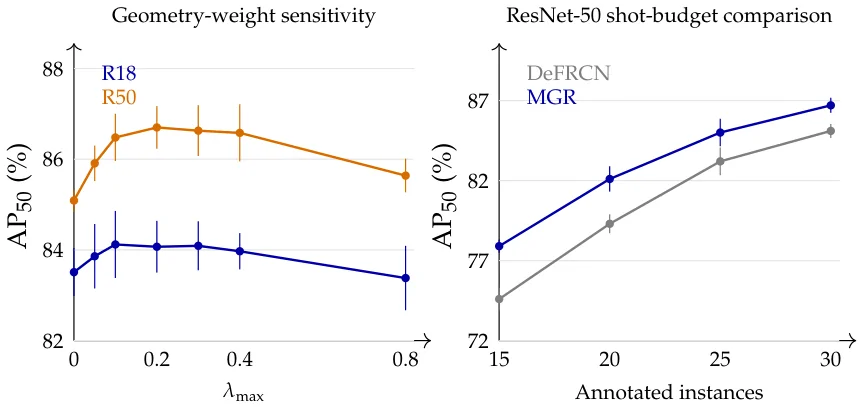
Geometry-weight sensitivity at 30 shots (**left**) and shot-budget comparison with ResNet-50 (**right**), corresponding to Table 6 and Table 17, respectively. Points show mean AP_50_ and error bars indicate one sample standard deviation across three runs. The curves visualize the reported aircraft-door experiments.

Table 17 shows a broad useful range rather than a uniformly optimal geometry weight. For ResNet-18, $\lambda_{\max}=0.10$ and 0.30 exceed the default 0.20 by only 0.05 and 0.02 AP_50_ points; these differences do not establish a reliable preference. Because $\lambda_{\max}$ scales all geometric terms, the results also do not isolate symmetry-specific tuning (Figure 3). Backbone-dependent weights would require validation-based selection, not selection of the highest test-set mean.

Sensitivity to the gate scale.

We vary σ∈{0.10, 0.15, 0.20, 0.25, 0.35, 0.50} for ResNet-18 and ResNet-50 at 30 shots, keeping support sets, paired runs, initialization, architecture, and training settings fixed with $\lambda_{\max}=0.20$. Each setting uses three runs; the default σ=0.25 corresponds to Full in Table 12 and Table 13.

**Figure 4:**
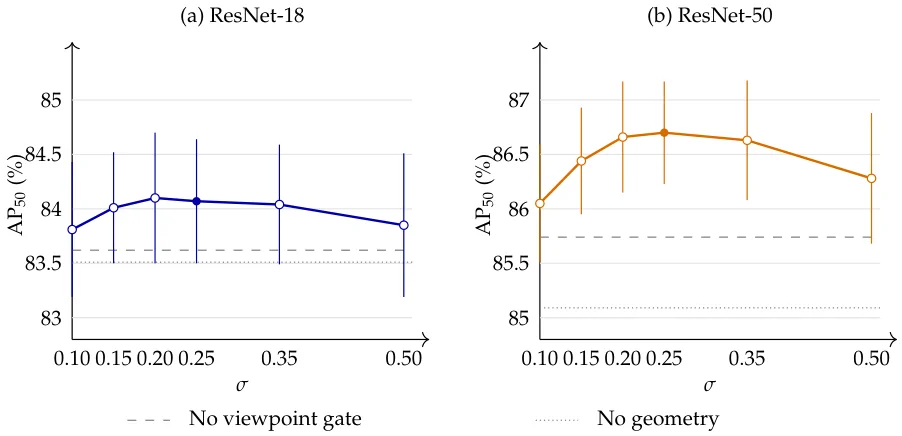
Gate-scale sensitivity at 30 shots with $\lambda_{\max}=0.20$ for (**a**) ResNet-18 and (**b**) ResNet-50. Points show means and error bars show sample SDs across three paired runs; filled markers identify the default σ=0.25. Dashed and dotted lines indicate the mean AP_50_ of No viewpoint gate and No geometry. Both panels use the same vertical span with different offsets.

**Figure 5:**
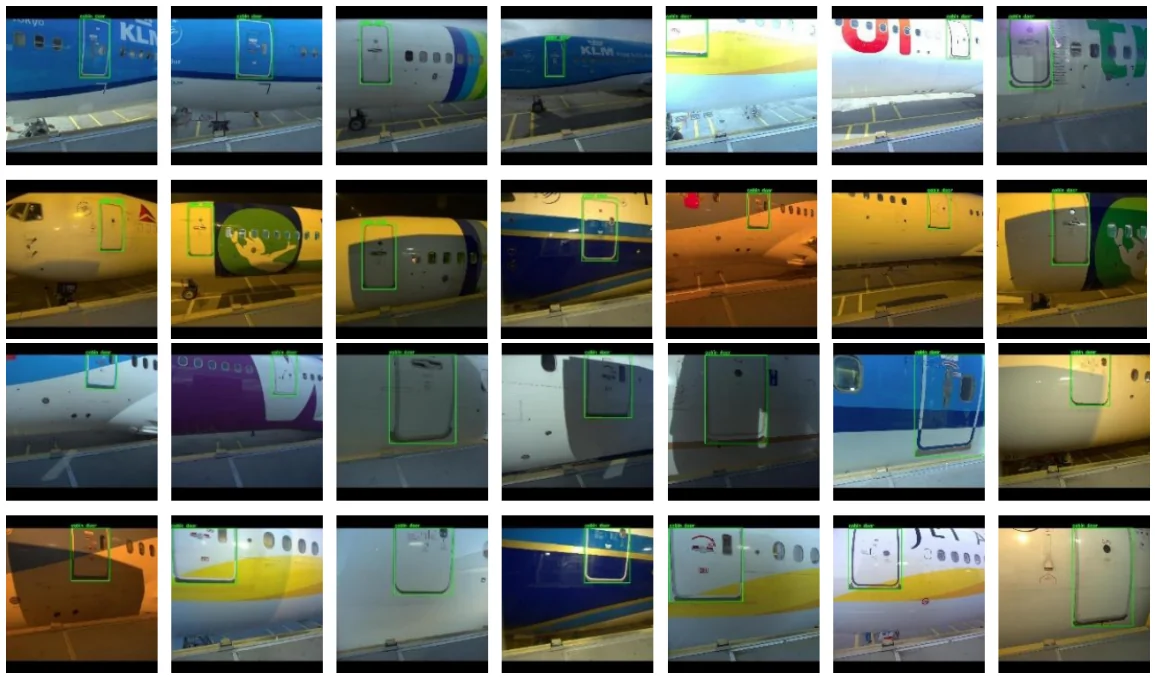
Qualitative aircraft-door detection examples under different illumination and occlusion conditions. Green rectangles indicate predicted door bounding boxes. The scene views preserve the surrounding context and illustrate detection behavior under different imaging conditions.

**Figure 6:**
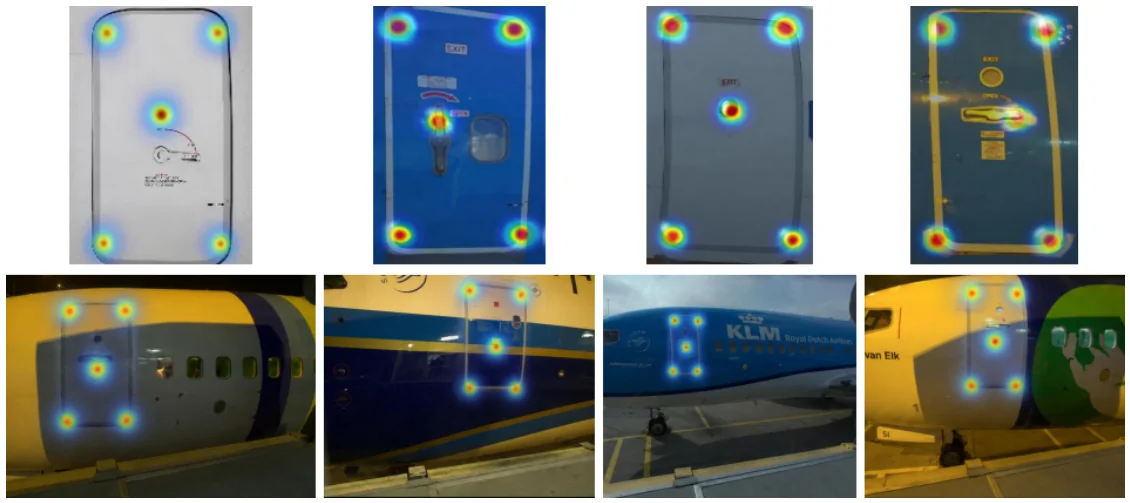
Qualitative landmark-response visualizations for aircraft doors at different image scales. Door-region views show the spatial distribution of landmark responses, while wider scene views retain the target scale and surrounding context.

**Figure 7:**
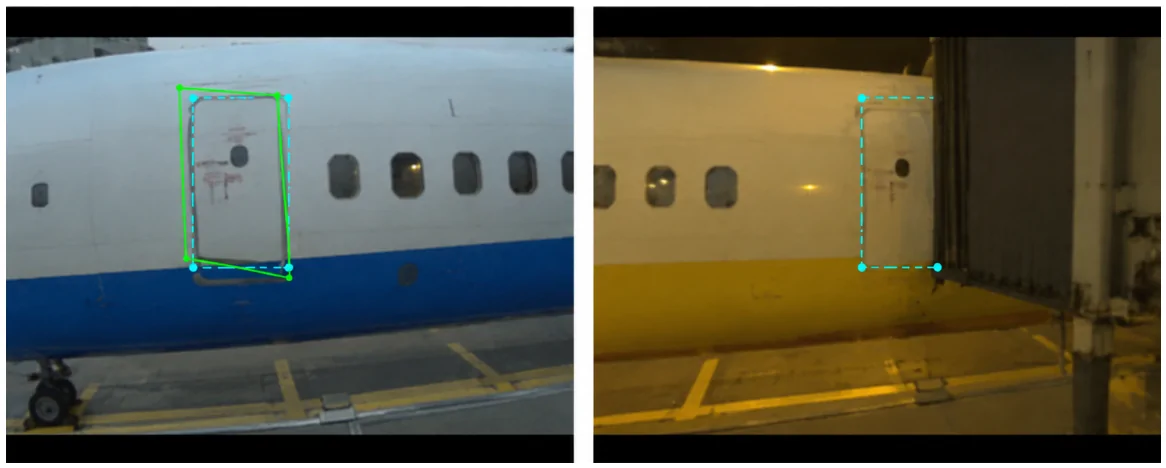
Failure examples from the test set. (**left**) Landmark localization error under an oblique view: predicted corner locations deviate from the valid ground-truth landmarks. (**right**) Missed detection under substantial occlusion: the annotated target is not matched by a retained detection under the stated evaluation rule. Cyan dashed boxes and cyan hollow markers indicate ground-truth boxes and valid landmarks; green boxes and filled markers indicate model predictions.

In Figure 4, the means form a broad plateau over σ=0.20–0.35. Across these three settings, AP_50_ ranges from 84.04 to 84.10 for ResNet-18 and from 86.63 to 86.70 for ResNet-50. Figure 5 and Figure 6 provide complementary qualitative views of detection and landmark responses. Figure 5 shows predicted boxes in their scene context, while Figure 6 illustrates the spatial concentration of landmark responses at different target scales. These overview visualizations are complemented by the enlarged failure examples in Figure 7, where ground-truth annotations and model outputs are compared directly.

Figure 7 (left) illustrates a limitation of landmark localization under perspective distortion. Image-space symmetry and orthogonality need not hold for an obliquely viewed door, and compatibility gating can attenuate these penalties without guaranteeing accurate corner placement. The example is consistent with this limitation, but does not establish that a particular geometric term caused the observed error.

Figure 7 (right) illustrates failure under substantial occlusion, where the visible appearance and frame structure provide insufficient evidence for a retained detection. Landmark and geometric losses supervise positive RoIs during training and cannot guarantee recovery of a missing detection at inference. Together, these examples distinguish geometric consistency from correct localization and successful detection.

## 5. Discussion

### 5.1. Major Discussion

The main comparisons show that MGR is competitive, with larger observed margins for ResNet-50 and mixed rankings for smaller backbones. This pattern suggests that the usefulness of structural supervision depends on the capacity of the accompanying appearance model, but the comparisons do not establish capacity as the sole cause. The paired confidence intervals limit the interpretation of small mean differences; numerical rankings alone do not demonstrate uniform superiority.

The two-factor ablation in Table 15 separates the conditional effects of refinement and geometry under identical landmark supervision. Refinement produces the larger observed increment, while geometry adds to both refined and unrefined configurations. Geometry is therefore the methodological focus because of its treatment of projectively valid and viewpoint-sensitive relations, not because it accounts for most of the performance gain. Comparisons against box-only models assess the complete architecture and its additional labels.

Geometric constraints must be interpreted according to their validity under projection. Incidence is preserved for a planar frame, while image-space symmetry and orthogonality depend on viewpoint. Masking and gating reduce unsuitable supervision but cannot eliminate all mismatch, as the oblique-view results and mixed symmetry ablations illustrate. Likewise, higher GCR need not mean better localization: an internally regular configuration may still miss the door. Independent center prediction supplies an auxiliary consistency target, not additional manual information or a universally necessary representation.

### 5.2. Limitations

The evaluation uses a private, single-category aircraft-door dataset, which limits independent verification and generalization to other object families. Sequence-level splitting reduces temporal leakage, but incomplete aircraft identity metadata limits verification of identity separation across partitions. The few-shot budget counts target-domain adaptation instances and does not include the separately labeled validation set; it therefore does not represent the total annotation expenditure. In addition, three paired runs provide limited precision for small performance differences and do not independently quantify support-set and optimizer variability.

Landmark annotation introduces additional effort and uncertainty, particularly where rounded corners require extrapolation or landmarks are partly occluded. The annotation-derived gate can consequently reflect labeling quality as well as viewpoint compatibility. Strong curvature, open-door configurations, truncation, and extreme foreshortening can violate the geometric assumptions. The uniform OKS tolerance is a task-specific evaluation choice rather than a landmark-wise tolerance calibrated from annotator variability. Finally, the additional prediction and refinement modules increase computational requirements relative to the box-only baseline. Image-space accuracy and single-device runtime evaluation are insufficient to establish metric docking accuracy or operational safety.

### 5.3. Future Research Directions

Future work should examine generalization across additional airports, aircraft appearances, and object categories, supported by shareable evaluation resources and reproducible sequence-level splits. More repeated runs and controlled variation of support sampling and optimization would improve uncertainty estimation. Evaluation under a joint adaptation and validation annotation budget would also provide a more complete assessment of data efficiency.

Methodological extensions include validation-based selection of backbone-dependent or term-specific regularization strengths, uncertainty-aware landmark supervision, and evaluation tolerances informed by inter-annotator variability. Geometry that accommodates non-planar or articulated door configurations may improve performance where the current assumptions are unsuitable. Reducing the cost of the landmark heads and RoI refinement is another practical direction. For deployment, the perception model should be integrated with calibrated metric localization and evaluated within the complete boarding-bridge system, including failure detection and recovery.

## 6. Conclusions

MGR combines landmark supervision and feature refinement with geometric regularization that distinguishes diagonal incidence from viewpoint-sensitive symmetry and orthogonality. It reaches 86.70% AP_50_ with ResNet-50 at 30 shots and an average observed margin of 1.10 percentage points over SGFNet across the four main budgets. Smaller backbones show mixed rankings. Relative to SGFNet, condition-specific means favor MGR for nighttime, occlusion, and the held-out airport, but not daytime or oblique views. Annotation-matched ablations show additional mean gains from geometry, while refinement contributes a larger increment in the ResNet-50 comparison. The results support geometry as a complementary training signal whose usefulness depends on capacity, annotation reliability, and imaging conditions; they do not establish uniform superiority or deployment-level safety.

## Figures and Tables

**Table 1:** Evaluation-subset statistics. Instances denote annotated aircraft doors; condition subsets can overlap.

Evaluation Subset	Images	Instances	Sequences	Airports
In-domain test, all	1200	1320	120	3
Day	780	858	78	3
Night	420	462	42	3
Occlusion	300	336	45	3
Oblique	240	264	36	3
Held-out airport test	360	396	36	1

**Table 2:** Annotation time in seconds for individual operations and support-set budgets.

Operation or Budget	Box-Only	MGR	Additional
Box annotation per instance	10	10	0
Four-corner annotation per instance	0	8	8
Validity and extra checking per instance	0	2	2
Independent center annotation	0	0	0
Total per instance	10	20	10
15-shot support set	150	300	150
20-shot support set	200	400	200
25-shot support set	250	500	250
30-shot support set	300	600	300

**Table 3:** Annotation audit and metric-constraint eligibility statistics. Coordinate disagreement is evaluated only for corners marked valid by both annotators.

Audit Item	Value
Independently reannotated door instances	300
Corner-label pairs inspected	1200
Corners valid for both annotators	1080
Validity-label agreement	96.5%
Median coordinate disagreement, original-image pixels	2.1
90th-percentile coordinate disagreement, original-image pixels	5.8
Median disagreement divided by $\sqrt{\mathrm{area}(b^{*})}$	0.010
90th-percentile normalized disagreement	0.029
Door instances across the four partitions	6006
Invalid corner labels/all corner labels	1922/24,024 (8.00%)
Instances with an invalid derived-center target	1021/6006 (17.00%)
Open-panel instances excluded from metric terms	180
Strongly curved instances excluded from metric terms	120
Overlap between the preceding two categories	30
Other excluded instances, disjoint from the preceding union	90
Total instances excluded from metric terms	360/6006 (5.99%)

**Table 4:** Matched-backbone AP_50_ (%) for ResNet-18. Method rows report mean ± SD; difference rows report paired mean differences and 95% CIs in brackets, in percentage points. Box-only uses box supervision; MGR also uses landmark labels.

Method	15-Shot	20-Shot	25-Shot	30-Shot
Meta R-CNN	65.72 ± 0.44	69.86 ± 0.81	73.91 ± 0.66	77.14 ± 0.49
TFA	68.92 ± 0.55	73.21 ± 0.65	77.36 ± 0.74	80.54 ± 0.61
FSCE	70.87 ± 0.95	74.76 ± 0.56	79.42 ± 0.43	82.36 ± 0.43
DeFRCN	72.11 ± 0.99	76.92 ± 0.37	81.37 ± 0.49	83.64 ± 0.76
SGFNet	72.94 ± 0.51	77.13 ± 0.58	81.65 ± 0.83	83.87 ± 0.85
MGR (Box-only)	70.25 ± 0.68	74.86 ± 0.59	79.15 ± 0.72	81.96 ± 0.54
MGR	72.82 ± 0.95	77.69 ± 0.45	81.28 ± 0.88	84.07 ± 0.57
MGR − SGFNet	−0.12 [−2.17, 1.93]	+0.56 [−0.75, 1.87]	−0.37 [−2.50, 1.76]	+0.20 [−1.66, 2.06]
MGR − DeFRCN	+0.71 [−1.70, 3.12]	+0.77 [−0.26, 1.80]	−0.09 [−1.99, 1.81]	+0.43 [−1.27, 2.13]
MGR − Box-only	+2.57 [0.46, 4.68]	+2.83 [1.50, 4.16]	+2.13 [0.11, 4.15]	+2.11 [0.73, 3.49]

**Table 5:** Matched-backbone AP_50_ (%) for ResNet-34. Method rows report mean ± SD; difference rows report paired mean differences and 95% CIs in brackets, in percentage points. Box-only uses box supervision; MGR also uses landmark labels.

Method	15-Shot	20-Shot	25-Shot	30-Shot
Meta R-CNN	67.13 ± 0.96	71.31 ± 0.49	74.62 ± 0.81	78.39 ± 0.50
TFA	70.47 ± 0.58	75.43 ± 0.66	78.94 ± 0.45	82.03 ± 0.86
FSCE	72.68 ± 0.55	77.31 ± 0.82	80.86 ± 0.46	83.64 ± 0.69
DeFRCN	73.88 ± 0.46	78.57 ± 0.79	82.93 ± 0.70	84.88 ± 0.55
SGFNet	74.92 ± 0.66	79.80 ± 0.61	83.78 ± 0.62	85.77 ± 0.56
MGR (Box-only)	72.48 ± 0.61	76.92 ± 0.73	80.74 ± 0.48	82.83 ± 0.62
MGR	75.16 ± 0.75	79.66 ± 0.43	84.02 ± 0.57	85.71 ± 0.39
MGR − SGFNet	+0.24 [−1.52, 2.00]	−0.14 [−1.49, 1.21]	+0.24 [−1.24, 1.72]	−0.06 [−1.30, 1.18]
MGR − DeFRCN	+1.28 [−0.35, 2.91]	+1.09 [−0.61, 2.79]	+1.09 [−0.51, 2.69]	+0.83 [−0.39, 2.05]
MGR − Box-only	+2.68 [0.96, 4.40]	+2.74 [1.16, 4.32]	+3.28 [1.96, 4.60]	+2.88 [1.53, 4.23]

**Table 6:** Matched-backbone AP_50_ (%) for ResNet-50. Method rows report mean ± SD; difference rows report paired mean differences and 95% CIs in brackets, in percentage points. Box-only uses box supervision; MGR also uses landmark labels.

Method	15-Shot	20-Shot	25-Shot	30-Shot
Meta R-CNN	68.40 ± 0.48	72.60 ± 0.69	75.10 ± 0.62	79.20 ± 0.45
TFA	71.80 ± 0.45	76.90 ± 0.35	80.10 ± 0.61	82.70 ± 0.62
FSCE	73.20 ± 0.92	78.00 ± 0.86	81.50 ± 0.78	83.60 ± 0.51
DeFRCN	74.60 ± 0.71	79.30 ± 0.59	83.20 ± 0.87	85.10 ± 0.44
SGFNet	76.10 ± 0.80	80.70 ± 0.80	84.50 ± 0.58	86.00 ± 0.36
MGR (Box-only)	73.36 ± 0.76	77.91 ± 0.58	81.84 ± 0.65	83.39 ± 0.46
MGR	77.90 ± 0.41	82.10 ± 0.79	85.00 ± 0.86	86.70 ± 0.47
MGR − SGFNet	+1.80 [0.08, 3.52]	+1.40 [−0.58, 3.38]	+0.50 [−1.39, 2.39]	+0.70 [−0.36, 1.76]
MGR − DeFRCN	+3.30 [1.77, 4.83]	+2.80 [1.03, 4.57]	+1.80 [−0.35, 3.95]	+1.60 [0.47, 2.73]
MGR − Box-only	+4.54 [2.90, 6.18]	+4.19 [2.43, 5.95]	+3.16 [1.23, 5.09]	+3.31 [2.15, 4.47]

**Table 7:** Runtime and peak allocated GPU memory for ResNet-50 at 30 shots on an RTX 3090.

Configuration	Inference ms/Image	Throughput Images/s	Inference Peak GiB	Training ms/Update	Training Peak GiB
Box-only baseline	52.0	19.23	1.92	184.0	4.80
No geometry	122.0	8.20	3.35	346.0	7.60
MGR	122.0	8.20	3.35	359.0	7.82

**Table 8:** Lower-budget comparison with ResNet-50.

Configuration	5-Shot	10-Shot	15-Shot
SGFNet	64.38 ± 1.42	71.83 ± 1.06	76.10 ± 0.80
No geometry	63.91 ± 1.36	71.36 ± 1.08	76.08 ± 0.73
MGR	65.24 ± 1.51	73.08 ± 1.12	77.90 ± 0.41

**Table 9:** Few-shot and full-training-pool references on the same in-domain test set. AP_50_ is reported as mean ± sample standard deviation across three runs. YOLOv5s is an architecture-specific reference rather than a matched-backbone comparison.

Configuration	Target Supervision	30-Shot	Full Pool: 3960 Instances
YOLOv5s	Boxes	82.76 ± 0.81	91.12 ± 0.35
MGR (Box-only), ResNet-50	Boxes	83.39 ± 0.46	91.58 ± 0.29
MGR, ResNet-50	Boxes and landmarks	86.70 ± 0.47	92.43 ± 0.33

**Table 10:** Condition-specific AP_50_ (%) at 30 shots with ResNet-50. Conditions overlap and are not averaged to obtain overall AP.

Condition	TFA	FSCE	DeFRCN	SGFNet	MGR
Day	88.91 ± 0.74	88.76 ± 0.52	89.31 ± 0.51	89.42 ± 0.48	89.08 ± 0.79
Night	79.83 ± 0.54	81.07 ± 0.69	82.48 ± 0.46	83.56 ± 0.61	84.37 ± 0.64
Occlusion	76.47 ± 0.56	77.86 ± 0.33	79.03 ± 0.67	81.24 ± 0.59	82.11 ± 0.74
Oblique	77.08 ± 0.55	80.96 ± 0.72	80.74 ± 0.57	81.18 ± 0.63	80.49 ± 0.68
Held-out airport	75.36 ± 0.75	76.84 ± 0.42	78.31 ± 0.71	79.72 ± 0.66	80.16 ± 0.70

**Table 11:** Offline gate diagnostics for positive RoIs with ResNet-50 at 30 shots and σ=0.25. Gate summaries and threshold fractions are conditional on valid configurations. Percentages and quantiles are computed within each run and averaged across three runs.

Condition	Valid Configurations (%)	Median $g^{*}$	90th Percentile $g^{*}$	$g^{*}<0.1$ (%)
Day	91.8	0.64	0.93	12.6
Night	86.4	0.59	0.91	16.8
Occlusion	63.7	0.53	0.89	21.4
Oblique	84.1	0.07	0.41	61.8
Held-out airport	85.3	0.46	0.88	26.7

**Table 12:** Single-factor ablations at 30 shots with ResNet-50. OKS is multiplied by 100.

Configuration	AP_50_	100 × OKS	GCR (%)
Full	86.70 ± 0.47	83.40 ± 0.56	92.30 ± 0.72
No geometry	85.09 ± 0.24	80.96 ± 0.39	87.62 ± 0.51
No multi-scale attention	85.61 ± 0.56	82.87 ± 0.55	92.01 ± 0.58
No landmark attention	85.88 ± 0.31	81.91 ± 0.43	91.14 ± 0.53
No incidence	86.21 ± 0.51	82.03 ± 0.47	90.16 ± 0.31
No symmetry	86.39 ± 0.44	83.11 ± 0.26	91.53 ± 0.75
No orthogonality	86.47 ± 0.38	83.49 ± 0.27	91.71 ± 0.72
No viewpoint gate	85.74 ± 0.45	82.36 ± 0.56	93.06 ± 0.29
No warm-up	86.31 ± 0.44	82.97 ± 0.49	91.98 ± 0.30
Keypoint baseline	83.26 ± 0.63	79.14 ± 0.47	85.63 ± 0.54

**Table 13:** Single-factor ablations at 30 shots with ResNet-18. OKS is multiplied by 100.

Configuration	AP_50_	100 × OKS	GCR (%)
Full	84.07 ± 0.57	80.52 ± 0.57	90.02 ± 0.66
No geometry	83.51 ± 0.53	79.44 ± 0.76	86.81 ± 0.75
No multi-scale attention	83.83 ± 0.58	80.16 ± 0.24	89.83 ± 0.74
No landmark attention	83.69 ± 0.74	79.81 ± 0.55	89.37 ± 0.35
No incidence	83.85 ± 0.47	79.72 ± 0.79	88.39 ± 0.28
No symmetry	84.11 ± 0.65	80.23 ± 0.68	89.58 ± 0.38
No orthogonality	83.96 ± 0.71	80.63 ± 0.34	89.66 ± 0.51
No viewpoint gate	83.62 ± 0.57	79.83 ± 0.41	90.83 ± 0.65
No warm-up	83.91 ± 0.62	80.14 ± 0.25	89.75 ± 0.37
Keypoint baseline	82.67 ± 0.63	78.13 ± 0.62	84.46 ± 0.75

**Table 14:** Precision and recall for selected 30-shot ResNet-50 configurations at a common score threshold.

Configuration	Precision (%)	Recall (%)
Full	87.60 ± 0.32	84.90 ± 0.32
No geometry	87.13 ± 0.41	83.14 ± 0.75
No multi-scale attention	87.69 ± 0.28	83.22 ± 0.48
No symmetry	87.79 ± 0.40	84.08 ± 0.42

**Table 15:** Two-factor ablation of refinement and geometric regularization with ResNet-50 at 30 shots. Refinement jointly denotes multi-scale aggregation and landmark-guided attention.

Configuration	Refinement	Geometry	AP_50_ (%)
Keypoint baseline	Off	Off	83.26 ± 0.63
Geometry only	Off	On	84.74 ± 0.58
No geometry	On	Off	85.09 ± 0.24
Full	On	On	86.70 ± 0.47

**Table 16:** Center-representation ablation with ResNet-50 at 30 shots. Results are reported as mean ± sample standard deviation across three paired runs.

Configuration	Heatmaps	Center	Incidence	AP_50_ (%)
Four-corner	4	Computed	Off	86.08 ± 0.58
No incidence	5	Predicted	Off	86.21 ± 0.51
Full	5	Predicted	On	86.70 ± 0.47

**Table 17:** Sensitivity to the maximum geometry weight at 30 shots.

$\lambda_{\max}$	ResNet-18 AP_50_	ResNet-50 AP_50_
0.00	83.51 ± 0.53	85.09 ± 0.24
0.05	83.86 ± 0.71	85.91 ± 0.39
0.10	84.12 ± 0.74	86.48 ± 0.52
0.20	84.07 ± 0.57	86.70 ± 0.47
0.30	84.09 ± 0.54	86.63 ± 0.56
0.40	83.97 ± 0.40	86.58 ± 0.63
0.80	83.38 ± 0.71	85.64 ± 0.37

## Data Availability

The relevant materials can be obtained by contacting the corresponding author, including the code and dataset links.

## Abbreviations

The following abbreviations are used in this manuscript:

AdamW	Adaptive moment estimation with decoupled weight decay
AI	Artificial intelligence
AP	Average precision
BL	Bottom left
BR	Bottom right
CBAM	Convolutional block attention module
CI	Confidence interval
COCO	Common Objects in Context
CUDA	Compute Unified Device Architecture
DeFRCN	Decoupled Faster R-CNN
DSNT	Differentiable spatial to numerical transform
FP32	32-bit floating-point precision
FPN	Feature pyramid network
FSCE	Few-Shot Object Detection via Contrastive Proposal Encoding
FSOD	Few-shot object detection
GAP	Global average pooling
GCR	Geometric consistency rate
GiB	Gibibyte
GMAC	Giga multiply–accumulate operations
GPU	Graphics processing unit
IoU	Intersection over union
MAC	Multiply–accumulate operation
MGR	Multi-constraint geometric regularization
MLP	Multilayer perceptron
NMS	Non-maximum suppression
OKS	Object keypoint similarity
R-CNN	Region-based convolutional neural network
ReLU	Rectified linear unit
ResNet	Residual network
RoI	Region of interest
RPN	Region proposal network
SD	Standard deviation
SGFNet	Structure-guided few-shot object detection network
SSD	Single Shot MultiBox Detector
TFA	Two-stage fine-tuning approach
TL	Top left
TR	Top right
YOLO	You Only Look Once

## Appendix A. Nomenclature

**Table A1:** Notation and implementation dimensions.

Symbol	Meaning
*I*	Input image.
$b,b^{*}$	Proposal bounding box and matched ground-truth box.
*K*	Number of annotated door instances in a support set.
$K^{*},\hat{K}$	Annotated and predicted landmark configurations.
$k_{i}^{*},{\hat{k}}_{i},{\tilde{k}}_{i}$	Annotated, predicted, and box-diagonal-normalized coordinates of landmark *i*.
*N*	Landmark count; N=5 in Full MGR and N=4 in the four-corner variant.
i,j,k	Landmark indices in the geometric formulation; *i* denotes a run index in the statistical analysis.
TL, TR, BR, BL, C	Top-left, top-right, bottom-right, bottom-left, and center landmark labels.
$v_{i},{\bar{v}}_{i}$	Annotation-validity mask and RoI-specific effective validity mask; *v* denotes the collection of annotation masks.
$d^{*}$	Ground-truth box diagonal in image pixels.
$R,R_{+}$	Number of processed RoIs and number of positive training RoIs.
*C*	Feature-channel count, 256; distinct from the center label C.
S,U	RoI feature-grid area, $14^{2}$, and heatmap-grid area, $56^{2}$.
*B*	Number of scale branches, 3.
*L*	Total loss in the objective; in complexity expressions, number of convolutions per landmark head, 4.
*D*	Channel-projection bottleneck dimension, 16; distinct from the distance function D(a;b,c).
$r,r^{\prime }$	Scale-branch indices associated with dilation rates 1, 3, 6; $r^{\prime }$ is a summation index.
*c*	Channel index in $a_{r,c}$ and $h_{c}$; a point argument in D(a;b,c).
$X,Z_{r},X_{m},X_{s}$	Original RoI features, branch features, scale-fused features, and spatially refined features.
$a_{r,c},a_{r}$	Channel-specific scale weight and its vector over channels.
$h,h_{c}$	Shared channel-projection function and its channel-*c* output; the superscript h∈{0, 1} separately denotes the landmark- head index.
$H_{i}^{h},P_{i}^{h}$	Heatmap logits and spatial probability distribution for landmark *i* in head *h*; 0 denotes coarse and 1 refined.
Ω,p,q	Heatmap lattice, coordinate vector, and dummy lattice coordinate in spatial sums or maxima.
τ	Spatial softmax temperature, 1.
A,γ	Landmark-attention map and residual modulation strength, 1.
ϵ	Numerical stabilizer, $10^{-6}$.
$Q_{i},\ell_{i}^{h}$	Normalized Gaussian target and distribution loss for landmark *i* in head *h*.
Gaussian target width	Standard deviation of 1.5 heatmap pixels.
D(a;b,c)	Stabilized squared distance from point *a* to the line through points b,c.
D	Set of center–diagonal landmark triples.
u,l	Upper-edge and lower-edge midpoints in normalized coordinates.
*a*	Stabilized direction vector from *l* to *u* in the symmetry expression; a point argument in D(a;b,c).
$x,R_{a}\left(x\right),I_{2}$	Generic two-dimensional point, its reflection mapping, and the 2×2 identity matrix.
e,f	Adjacent directed edge vectors along the ordered corner polygon.
$E_{\mathrm{sym}},E_{\mathrm{orth}}$	Ungated symmetry and orthogonality residuals.
$q^{*},g^{*},\sigma$	Annotation-derived compatibility residual, gate value, and gate scale, 0.25.
$L_{\mathrm{inc}},L_{\mathrm{sym}},L_{\mathrm{orth}}$	Masked incidence loss and gated symmetry and orthogonality losses.
$L_{\mathrm{kp}},L_{\mathrm{geo}}$	Landmark-supervision loss and combined geometric loss.
$L_{\mathrm{RPN}},L_{\det}$	Proposal-network loss and RoI detection loss.
$t,T,T_{w}$	Training-update index, total update budget, and geometry warm-up length, 200.
$\lambda \left(t\right),\lambda_{\max}$	Update-dependent geometry weight and its final value, 0.20.
$\theta ,\eta_{t}$	Trainable model parameters and learning rate at update *t*.
$C_{\mathrm{base}},C_{\mathrm{extra}},C_{\mathrm{total}}$	Base-detector, additional-pathway, and total forward- operation counts.
$P_{\mathrm{extra}}$	Additional learned-weight count excluding biases.
$s,\kappa_{i}$	Square root of ground-truth box area and OKS tolerance, 0.05.
$d_{i},\bar{d},s_{d}$	Run-level AP difference, its sample mean, and its sample standard deviation.
$n,t_{0.975,n-1}$	Number of paired runs, 3, and the 97.5th percentile of Student’s *t* distribution with n−1 degrees of freedom.
